# Rapid spread of the emerging cosmopolitan genotype of dengue virus serotype 2, and expansion of dengue virus serotype 1 genotype V in Peru

**DOI:** 10.17843/rpmesp.2024.414.13898

**Published:** 2024-12-02

**Authors:** Henri Bailon, Víctor Jimenez, Marco Galarza, Princesa Medrano, Orson Mestanza, Dana Figueroa, Wendy Lizarraga, Iris Silva, Luren Sevilla, Verónica Hurtado, Vanessa Izarra, Carlos Padilla, Luis Barcena, Omar Caceres, Susy Merino, Adolfo Marcelo, Nora Ruiz, Hapuarachchige Chanditha Hapuarachchii, César Cabezas Sánchez, María P. García

**Affiliations:** 1 Innovation and Development Area (Genomic Surveillance Team), National Center for Public Health (CNSP), National Institute of Health (INS), Lima, Peru. Innovation and Development Area (Genomic Surveillance Team) National Center for Public Health (CNSP) Instituto Nacional de Salud (INS) Lima Peru; 2 National Referral Laboratory for Metaxenic Diseases and Viral Zoonoses, CNSP-INS, Lima, Peru. National Referral Laboratory for Metaxenic Diseases and Viral Zoonoses CNSP-INS Lima Peru; 3 National Referral Laboratory for Immunopreventable Viruses, CNSP-INS, Lima, Peru. National Referral Laboratory for Immunopreventable Viruses CNSP-INS Lima Peru; 4 Division of Microbiology and Molecular Epidemiology, Institute of Environmental Health, National Environment Agency, Singapore. Division of Microbiology and Molecular Epidemiology Institute of Environmental Health National Environment Agency Singapur; 5 Instituto Nacional de Salud, Lima, Peru. Instituto Nacional de Salud Lima Peru

**Keywords:** Peru, Dengue Virus, Genotype, E-glycoprotein, Dengue virus type 1, Dengue virus type 2

## Abstract

**Objectives.:**

This study aimed to evaluate the prevalence and distribution of dengue virus genotype V serotype 1 (DENV-1) and cosmopolitan genotype serotype 2 (DENV-2) in Peru between 2019 and 2022.

**Materials and methods.:**

The envelope (E) gene region was amplified from 79 serum samples by polymerase chain reaction (PCR) and sequenced by next-generation sequencing (NGS) technology. The obtained sequences were subsequently analyzed with bioinformatics tools.

**Results.:**

The study generated envelope gene sequences of DENV-1 and DENV-2 serotypes. Our study revealed a rapid dispersal and wide distribution of the cosmopolitan DENV-2 genotype in several regions of Peru in 2022, as well as the spread of DENV-1 genotype V to new Peruvian regions, along with the cosmopolitan DENV-2 genotype.

**Conclusions.:**

Our findings suggest the urgent need to strengthen epidemiological and genomic surveillance systems to understand and control the spread of circulating DENV genotypes in Peru. This will allow a more rapid response, as well as the monitoring of its potential dissemination to other countries in the Americas.

## INTRODUCTION

Dengue virus (DENV) is an RNA virus belonging to the Flaviviridae family and includes four serotypes, DENV-1, DENV-2, DENV-3 and DENV-4. DENV causes a febrile illness (dengue fever) and is classified as non-alarm and severe, and in certain cases can be fatal. Infection with a particular serotype of DENV results in lifelong immunity only against that specific serotype, therefore the risk of reinfection with a different serotype remains. These secondary DENV infections are known to be more severe than primary infections ^1^.

The countries of Central and South America, as well as those of the Caribbean, have a high annual burden of dengue, which has been increasing over the years.

Dengue has been of great public health importance since it was reported in Peru in the 1990s ^2^. The incidence of dengue in Peru has increased over the years due to the presence of *Aedes aegypti*, the main vector of DENV. All four serotypes of DENV have been present in Peru since its emergence. However, the occurrence and prevalence of specific serotypes and their associated genotypes have varied over time.

Peru has the second highest burden of dengue in the Americas, after Brazil. In 2022, 47,656 cases were reported, of which 87.4% were dengue without alarm signs, 12.3% were dengue with alarm signs, and 0.3% were severe dengue, resulting in a total of 65 deaths. In comparison, the country reported 21,407 cases with an incidence rate of 64.17 cases per 100,000 population during the same period in 2021 ^3^. The highest number of cases reported in 2022 occurred in the Peruvian regions of Loreto, Ica, Ucayali, Cusco, Cajamarca, San Martín, Lambayeque, Madre de Dios, Huánuco, Ancash, and Junín ^4^.

Peru’s ecology, climate and biodiversity provide an environment conducive to *Aedes aegypti* reproduction. The ubiquity of such habitats has led to increased vector dispersal. Among the four DENV serotypes, DENV-1 (particularly genotype V) has been the most frequently reported in several departments of Peru, with cases reported in Iquitos since the 1990s; DENV-2 (American genotype) was later introduced into Peru. The American/Asian genotype of DENV-2 (lineage II) emerged in late 2010 in Peru, causing many outbreaks and a nationwide dengue epidemic ^4^. The cosmopolitan genotype of DENV-2 was first reported in Peru during an outbreak in 2019 ^5^. It subsequently spread to different departments, including Piura, Lambayeque, Ica, Madre de Dios, Ancash, Cajamarca, San Martín, Loreto, Huánuco, and Ucayali, in 2022 ^6^. In 2021, Brazil also reported a case infected with the cosmopolitan genotype of DENV-2, indicating its likely spread to other neighboring countries ^7^. Consequently, countries in the region were advised to intensify their genomic surveillance efforts.

Therefore, it is crucial to have effective DENV surveillance in Peru to monitor the spread of new circulating genotypes, such as the cosmopolitan genotype. This is because its rapid geographic spread has been associated with an increase in the number of dengue cases. This study aimed to identify the prevalence and distribution of DENV-1 genotype V and DENV-2 cosmopolitan genotype in Peru using next-generation sequencing (NGS) technology and envelope gene-based phylogenetic analysis. In this study, we reveal an expansion of the pre-existing DENV-1 genotype V in Peru and a rapid spread of the DENV-2 cosmopolitan genotype in 2022 to different regions of Peru following its introduction in 2019.

**KEY MESSAGES**

Motivation for the study. This study aimed to analyze the prevalence and distribution of the V genotypes of dengue virus type 1 (DENV-1) and the cosmopolitan genotype DENV-2 in Peru, in order to understand their expansion in different regions; since these viruses can cause important outbreaks in the country.

Main findings. A rapid spread and wide distribution of the cosmopolitan DENV-2 genotype was reported in Peru in 2022, following its initial introduction in 2019. DENV-1 genotype V, present in Peru since the 1990s, has now expanded to new Peruvian regions, after previously being restricted to rural and jungle areas in the north of the country.

Implications. The results highlight the urgent need to strengthen epidemiological and genomic surveillance systems to monitor and control the spread of these genotypes in Peru, allowing a more rapid response and controlling their potential spread to other regions of the Americas.

## MATERIALS AND METHODS

### Sample data

The study analyzed 79 dengue-positive samples collected in Peru by the National Institute of Health as part of the diagnosis and surveillance of viral metaxenic disease arboviruses. In addition, the Center for Disease Control and Prevention (CDC) of Peru provided epidemiological information on circulating serotypes of DENV from 2019 to 2022.

### Extraction of genetic material and sequencing

Viral RNA was extracted from human serum using the MagaBio plus III viral DNA/RNA purification kit (BioFlux) following the manufacturer’s recommended protocol. Subsequently, the DENV envelope gene (E gene) was amplified by reverse transcription-polymerase chain reaction (RT-PCR) technology using the Superscript III Platinum one-step RT-PCR kit (Invitrogen™) and specific primers. The amplified products were purified with the Mag-Bind Total Pure NGS kit (Omega Bio-Tek) and sequenced with the Nextera XT kit (Illumina) on the MiSeq genomic sequencer (Illumina).

### Sequence data processing and phylogenetic analysis

The read quality of the sequences was assessed using the FASTQC v.0.11.9 tool (https://www.bioinformatics.babraham.ac.uk/projects/fastqc/). Then, sequences of good quality (Q quality value greater than 20) were assembled with the NCBI reference E gene sequence of DENV-1 (NC_001477.1) and DENV-2 (NC_001474.2) using the programs BWA v.0 .0.17, samtools v.1.14 and IVAR v.1.3.1. Aliview editor v.1.28 and integrating genomic viewer (IGV) v.2.16.0 were used to manually curate the consensus sequences.

The complete E gene sequences of DENV-1(8) (n=1,338) and DENV-2 (n=1,521) were retrieved from the NCBI virus database for phylogenetic analysis. In addition, 14 DENV-2 positive samples collected during the December 2019 and January 2020 dengue outbreaks in Peru were also included. The MAFFT v7.475 program was used to perform multiple sequence alignment. The Maximum Likelihood (ML) method, implemented in the RAxML v8.2.10 program, was used to perform the phylogenetic analyses. The resulting ML tree was visualized with Microreact (https://microreact.org/).

### Bayesian analysis

*Bayesian phylogeny*

A time-scale Bayesian phylogeny was constructed for DENV-2 using the Bayesian Evolutionary Analysis by Sampling Trees (BEAST) v1.10.4 software package ^9^. The dataset consisted of 202 complete DENV-2 E gene sequences, including 125 Peruvian sequences and 77 global sequences retrieved from the NCBI database. The temporal signal of the final data set for each serotype was checked using TempEst version 1.5.3 ^10^. The jModelTest program ^11^^)^ was used to determine that the General Time Reversible (GTR+G4+I) surrogate model was the best fit. To avoid assuming any particular demographic scenario, an uncorrelated log-normal relaxed clock and Bayesian Coalescent Skyline Plot prior (10 steps) were used to construct the time-scaled tree. Markov Chain Monte Carlo (MCMC) analysis ^12^^)^ was run for 100 million generations, with a sample taken every 10,000 states. The output log files were visualized with Tracer v.1.5 ^13^. An effective sampling size (ESS) >200 was considered sufficient for parameter convergence. The maximum cladistic credibility (MCC) tree was constructed after removing the first 10% of all trees (burn-in) using TreeAnnotator v.1.7.4. The MCC tree was plotted in FigTree v.1.4.3 (http://tree.bio.ed.ac.uk/software/figtree/).

*Effective population size estimates*

The temporal variation of the effective population size (EPS) and divergence times were determined using the Bayesian method implemented in BEAST v1.10.4 software. Two independent MCMC runs were performed, each of 50 million generations with sampling every 5000 generations. The default a priori parameters were used. Bayesian analysis was calibrated over time and included the skyline coalescent model, as well as the strict and uncorrelated molecular clock models, and the GTR+I+G4 substitution model. In order to determine statistically robust convergence, we used TRACER v1.7.1 (http://tree.bio.ed.ac.uk/software/tracer/) with ESS values greater than 200 considered robust. The remaining data were combined in Log Combiner v1.10 after a 10% burn-in period. A summary tree with temporal calibration was generated using TreeAnnotator v.1.10 (https://beast.community/treeannotator) and visualized in FigTree v1.4.

*Lineage classification of the cosmopolitan DENV-2 genotype*

Lineage classification of the cosmopolitan genotype was performed by constructing a Neighbour Joining phylogenetic tree using the Kimura-2 parameter substitution model with gamma parameter and invariant sites (Г5 + I) in the MEGA 7 software package (https://academic.oup.com/mbe/article/33/7/1870/2579089). The analysis comprised 3,827 complete sequences of the E gene of the cosmopolitan DENV-2 genotype, including sequences from the study and those obtained from the NCBI database. The tree was visualized and annotated with FigTree v.1.4.3 (http://tree.bio.ed.ac.uk/software/figtree/).

### Ethical considerations

The study was not approved by an ethics committee because the data was obtained from the surveillance activity of the laboratory and not from a specific research. Nevertheless, the data were processed in accordance with the institution’s data processing standards and policies.

## RESULTS

Dengue cases tend to increase in Peru during the spring and summer seasons each year. According to data from the Peruvian Center for Disease Control and Prevention, the highest number of dengue cases occurred between weeks 14 and 18 of 2022 (supplementary material S1). In addition, cumulative graphs show a significant increase in dengue cases from 2019 onwards (supplementary material S1).

Peru has only detected DENV-1 (genotype V) and DENV-2 (Asian American and cosmopolitan genotypes) among the four DENV serotypes since 2019 (Table 1). The geographic distribution and serotype composition of the 79 DENV-positive samples tested in this study are summarized in Table 2.

**Table 1:** Introduction and circulation at different times of DENV serotypes/genotypes in Peru.

Serotype	Year of entry into Peru	Circulating genotypes up to 2019	Circulating genotypes up to 2022
DENV-1	1990	Genotype III	
		Genotype V	Genotype V
DENV-2	2001/2010	America/Asia	
		America	
	2019	Cosmopolitan	Cosmopolitan
DENV-3	2001	India-Genotype III	
DENV-4	2001	Indonesia-Genotype II	

**Table 2:** Distribution of the 79 positive dengue cases included in this study.

Departament	Region	DENV1/V		DENV2/C	
		2021	2022	2021	2022
Piura	Coast	-	1	-	-
Lambayeque		-	2	-	1
Ancash		-	3	-	-
La Libertad		-	-	2	-
Lima		-	5	3	7
Ica		1	1	-	-
Cajamarca	Sierra	-	3	2	3
Huánuco		-	-	2	3
Junín		1	2	2	3
Pasco		-	-	1	-
Ayacucho		-	-	2	2
Puno		1	-	-	-
Cusco		-	1	1	3
Loreto	Jungle	-	3	1	2
San Martin		1	-	1	-
Ucayali		2	2	-	2
Madre de Dios		-	-	2	1
Amazonas		-	-	4	

DENV1-V: Dengue Virus serotype 1, genotype V

DENV2-C: Dengue virus serotype 2, cosmopolitan genotype

**Figure 1:**
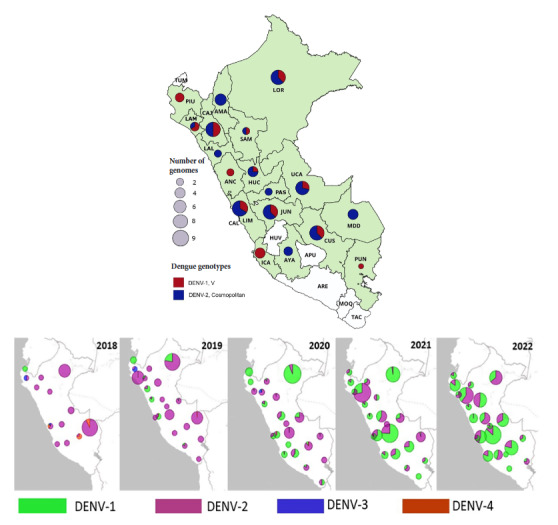
Frequency and distribution of Dengue infections in Peru by serotype and genotype from 2018 to 2022. Panel A shows the frequency of Dengue infections in Peru by serotype and genotype in 2022. The frequency distribution of each serotype is represented by a different color. Panel B shows the geographic distribution of DENV-1 (genotype V) and DENV-2 (cosmopolitan genotype) in Peru during the period 2018-2022.Dengue genotypesNumber of genomes.

Our results show that the cosmopolitan DENV-2 genotype was the most frequent among the study samples, representing 63.3% of the samples collected in the different regions of Peru. The cosmopolitan DENV-2 genotype caused the highest number of cases in Lima, but was also found in other regions such as Amazonas, Ayacucho, Cajamarca, Cusco, Huánuco, Junín, Loreto, Lambayeque, La Libertad, Madre de Dios, Pasco and Ucayali. Additionally, DENV-1 genotype V co-circulated in the regions of Lima, Loreto, Cuzco, Ucayali, San Martín, Cajamarca and Lambayeque (Table 2 and Figure 1).

In 2021, there were more cases of cosmopolitan genotype DENV-2 than genotype V DENV-1 in the jungle and mountain regions. Coastal regions reported few or no cases of these two genotypes. In 2022, both genotypes showed greater spread, with the cosmopolitan DENV-2 genotype expanding more in the jungle and mountain regions, and the V DENV-1 genotype expanding in the coastal regions (Table 2). The dengue burden in Peru in 2022 was driven by both DENV-1 genotype V and the cosmopolitan DENV-2 genotype, which expanded rapidly in the Peruvian coastal regions in 2021 and 2022.

Nonsynonymous mutations in the *E* gene of the cosmopolitan DENV-2 genotype include: M6I; V15I; Q52H; E71A; F119L; R120T; V129I; V140A; H149N; T180I; N203S; P222S; T226I; S255P; V309A; D329E; I380V; N390S; V428M; F429L; I462V; T478S and I484V, with all mutations highlighted in bold common to all sequences (Supplementary Material: GISAID accession cod. GISAID accession code and DENV gene E mutations).

**Figure 2:**
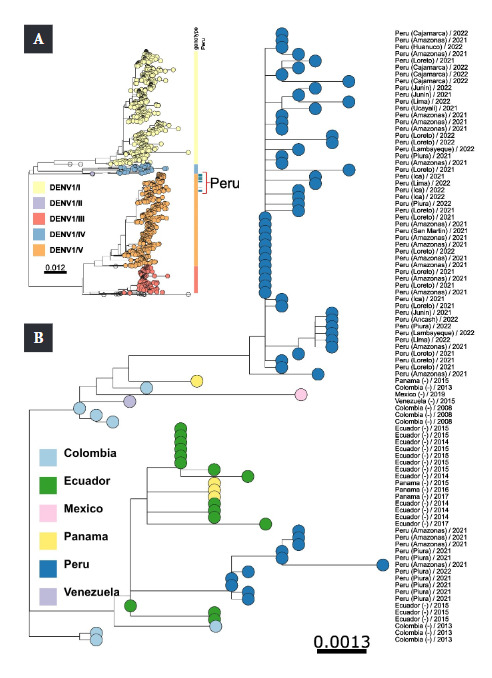
Maximum likelihood phylogenetic tree of DENV-1. Panel 3A shows the global phylogeny with Peruvian samples highlighted in blue bars and genotypes shown in different colors. Panel 3B shows a detailed phylogenetic sub-tree with the Peruvian samples represented by blue circles. Estimated changes by site are represented by horizontal bar scales.

The sequences of DENV-1 genotype V are genetically related to those of countries in the region, such as Ecuador, Colombia, Venezuela and Mexico. Two distinct clades were found within the DENV-1 genotype V from Peru. The dominant clade included samples from almost all Peruvian regions, particularly the northern and central regions, and was related to sequences from Ecuador (Figure 2A). The second cluster comprised samples from a few regions of southern Peru (Figure 2B). The initial detection of the dominant cluster in Lima and Ica in 2021, before its appearance in other regions in 2022, suggested a possible dispersal route from the central coast to the northern and southern regions of Peru. Similarly, the less dominant cluster was first detected in the central region of Junín in 2021, before emerging in Cusco, Ancash, Ucayali and Puno (Figure 2B).

DENV-1 genotype V had the following amino acid substitutions in the *E* gene : D317N; N52D; T88A; I114L; T161I; K203E; I293T; M297V; S338L; S339T; A369T; V345L; T368A; I394L; I436V; T441I; K483E; I439V; I457V; I573T; S619T; A649T; I716V; I719V; V482I and M484I, with mutations highlighted in bold, being common in all samples analyzed (Supplementary Material S2).

**Figure 3:**
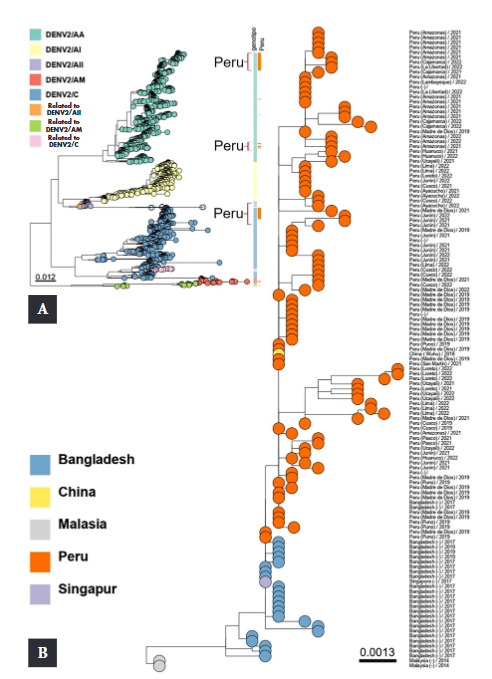
Maximum likelihood phylogenetic tree of DENV-2. Panel A shows the global phylogeny with Peruvian samples highlighted in orange bars and genotypes shown in different colors. Panel B shows a detailed phylogenetic sub-tree with the Peruvian samples in orange circles. Estimated changes by site are represented by horizontal bar scales.

Three genotypes have been identified since the first appearance of DENV-2 in Peru: American, Asian American (AA), and Cosmopolitan. The cosmopolitan DENV-2 genotype that has recently emerged in Peru is genetically related to those previously reported in Bangladesh ^5^. The first cases of the cosmopolitan DENV-2 genotype in Peru were reported in December 2019 in the Madre de Dios, Puno, and Cusco regions ^5^. These cases were also genetically related to those found in Bangladesh. In 2021, cases were reported in Madre de Dios, Amazonas, Junín, Cajamarca, Pasco, Ucayali, Loreto, Cusco, Ayacucho, Huánuco, and San Martín. The cosmopolitan genotype was the only circulating DENV-2 genotype in all regions of the country in 2022 (Figure 3B).

**Figure 4:**
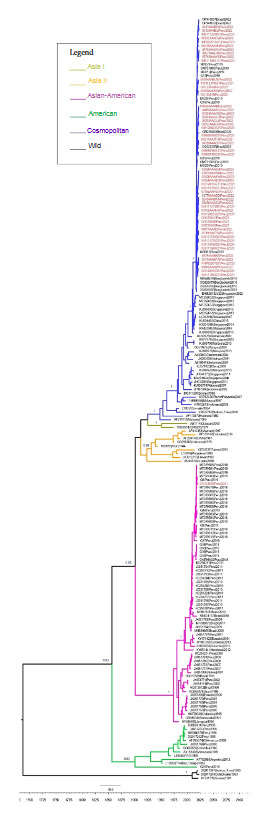
Dominance of DENV-2 genotypes in Peru before and after 2019. The time-scaled tree was constructed using the BEAST 1.10.4 software package, employing a general time-reversible substitution model (GTR+G4+I), a lognormal uncorrelated relaxed clock model, and an a priori Bayesian skyline tree. The Markov Chain Monte Carlo chain length was 100 million, with trees sampled every 10,000 iterations. Analyses included 202 complete envelope gene sequences.

Our findings show that the cosmopolitan genotype was dominant after 2019 for serotype 2; as the highest number of dengue cases was caused by the cosmopolitan genotype in all regions of Peru where cases occurred in 2021 and 2022 (Figure 4).

Our study revealed that cosmopolitan genotype lineage 5 was the dominant DENV-2 lineage in 2021- 2022. This finding coincides with a report in Brazil in November 2021^14^.

Analysis using the Bayesian skyline model showed a stable mean effective population size of DENV-1 genotype V in the long term, with a slight increase before 2020. On the other hand, the effective population size of the cosmopolitan DENV-2 genotype showed a rapid increase after its emergence in Peru in 2019 (supplementary material S3).

## DISCUSSION

This study evidences the rapid spread of dengue virus serotype 2 (DENV-2) cosmopolitan genotype and the expansion of dengue virus serotype 1 (DENV-1) genotype V in Peru between 2019 and 2022. Since its introduction in 2019, the cosmopolitan DENV-2 genotype has predominated in many regions, spreading rapidly and replacing previous genotypes such as Asian American. Likewise, the circulation of DENV-1 genotype V, present since the 1990s in jungle and rural areas, also showed a pattern of expansion into new areas. These findings highlight the urgent need to strengthen epidemiological and genomic surveillance to control the spread of these genotypes and assess their potential for spread in the Americas.

Dengue has been endemic in Peru for several years and affects almost all regions of the country. DENV transmission is favored by several factors, such as temperature, rainfall, distribution of mosquito vectors, water reservoirs, and poor housing conditions ^15^. The different regions of Peru are home to a wide variety of mosquitoes; the most prevalent species being *Aedes aegypti*, as well as some species of the *Anopheles* and *Culex* genera, which are found mainly in forested regions ^16^.

Dengue virus serotype 1 was first identified in 1990, and its genotypes III and V have been circulating from 2019 to 2022 ^17^. In 2010, DENV 2 Asian American genotype lineage II emerged and spread rapidly through many Amazonian cities and other regions of Peru, displacing the predominant serotype in those Amazonian areas, which was DENV-4; it also displaced DENV -2 Asian American genotype lineage I, but which had few cases ^18^^,^^19^.

Despite its high public health importance, dengue has received less priority since 2019 due to the high attention and resources devoted to COVID-19. This coincided with the emergence and wide dispersal of cosmopolitan genotype DENV-2, along with genotype V DENV-1 in Peru. The study revealed that cosmopolitan genotype lineage 5 has been present in Peru since 2019. This lineage has also been detected in Brazil, indicating its further spread to other South American countries ^20^.

Bayesian analysis showed a stable mean effective population size for DENV-1 genotype V over a long period of time, in contrast to the rapid increase in the effective population size of the cosmopolitan genotype following its emergence in 2019. Further research is required to determine whether the higher rate of expansion of the cosmopolitan genotype is due to a greater fitness advantage compared to the pre-existing American/Asian genotype and DENV-1 genotype V. This will help determine the epidemic potential of the cosmopolitan genotype of DENV-2 in Peru. In addition to its epidemic potential, the emergence of a new genotype may also result in changes in the severity of clinical cases. This may be influenced by comorbidities, infection status, population exposure to different serotypes, genetic variability, patient age, accessibility to services, and experience and quality of clinical management of patients ^21^^,^^22^.

Our study showed that the cosmopolitan DENV-2 genotype had a wide geographic distribution during the summer of 2022, indicating its rapid spread since the first report of a few cases in December 2019 ^5^. Given the natural history of dengue epidemics in Peru since the 1990s, the results highlight the importance of improving surveillance and outbreak risk assessment strategies to guide effective vector control measures and minimize the occurrence of dengue outbreaks. To determine the association between the occurrence of different DENV genotypes and the burden of dengue in Peru, it is necessary to complement the clinical-epidemiological information with genomic data of the virus. Table 2 and Figure 2 show the origin of 79 samples. Of these, 29 samples correspond to DENV-1 genotype V reported in the departments of Ancash, Ica, Piura and Puno. The remaining 50 samples are of the cosmopolitan DENV-2 genotype and were collected in the departments of Amazonas, Ayacucho, La Libertad, Madre de Dios and Pasco. Both DENV-1 genotype V and DENV-2 cosmopolitan genotype have been detected in some departments of Peru. These departments include Cajamarca, San Martín, Junín, Cusco and Lima. There are several factors that contribute to DENV transmission in these regions. Reported cases of dengue are usually found in subtropical and tropical regions, although some cases have also occurred in Puno. This may be due to geographic distribution and/or climatic changes in the different regions, as well as the presence of the mosquito vector *Aedes aegypti*^6^. Our findings are comparable to those by Mollinedo *et al*. (2021), who reported that the high population movement of mosquitoes across various ecological zones in subtropical and tropical climates is one of the factors contributing to the increased incidence of DENV cases in Bolivia. The study identified cases of DENV-1 and DENV-2, as well as circulating serotypes of DENV-3 and the first case of DENV-4 ^23^.

Real *et al*. ^24^^)^ reported an increase in dengue cases in Ecuador in recent years. The study conducted between 2000-2015 in the province of Guayas revealed the presence of the four circulating serotypes, with more cases caused by DENV-1 genotype V and DENV-2 American-Asian genotype. As for DENV-1, the Peruvian cases correspond to genotype V, which has been widely distributed in different departments of Peru. In addition, it was found that the genetic sequences of several Peruvian departments are evolutionarily related to sequences from other countries where DENV-1 genotype V has been reported ^25^.

Phylogenetic analysis of DENV-1 grouped the samples into two distinct clades. The first cluster is dominant and includes samples from almost all departments of Peru. The second cluster comprises cases belonging to DENV-1 genotype V from the years 2021 and 2022, from the southern and northern regions of Peru. The temporal relationship between the clusters is not clear. These results are similar to those reported by Ocasionez R. *et al*. ^26^, who found that the strains in Santander, Colombia corresponded to genotype V. The strains were compared with those from neighboring countries, such as Peru, Venezuela and Brazil, which also showed circulation of other serotypes.

Rojas *et al*. conducted a molecular study of dengue in Paraguay and found that the population of Asunción has experienced outbreaks since 1988, when DENV-1 was introduced into the country ^27^. This study characterized the virus as endemic, and reports that the DENV-1 serotype has the highest prevalence of dengue and co-circulates with DENV-2. These results coincide with ours.

Overall, the sequences of DENV-1 genotype V from Peru are genetically related to those from Ecuador, Colombia, Venezuela and Mexico reported between 2008 and 2019. This demonstrates the dispersion of this genotype in different regions of Peru and its evolutionary genetic relationship with other countries. Similarly, phylogenetic analysis of DENV-2 revealed that the cosmopolitan genotype was also dispersed in several regions of Peru.

Cruz C. *et al*. conducted research on the molecular epidemiology of DENV-2 in Peru, and their results suggest that the American genotype was introduced to Peru before 2001, and that the Asian American genotype has been circulating in Peru from 2000 onwards ^28^, with two lineages possibly introduced from the north through neighboring countries, such as Ecuador, Colombia, and Venezuela, and from the east through Brazil and Bolivia.

These studies show that earlier genotypes were circulating in Peru. As of 2022, the cosmopolitan genotype is the predominant genotype in Peru, with a notable absence of other genotypes. Phylogenetic analysis of DENV-2 cases suggests that after the appearance of the cosmopolitan genotype in the Madre de Dios region in 2019, it subsequently spread to other regions of Peru, with the Peruvian regions of Junín and Amazonas possibly being the potential centers of its early transmission.

Giovanetti *et al*. ^29^ studied the emergence of the cosmopolitan genotype of DENV-2 in Brazil and found that the strain obtained from a patient in Goiás is phylogenetically related to cases in Peru and Bangladesh; suggesting the onset of regional spread in the Americas. Susuki K. *et al*. ^30^ found two distinct lineages of DENV-2 in Bangladesh within the cosmopolitan genotype. These lineages are closely related to strains from the Philippines, Malaysia, and Singapore and have experienced increased genetic diversity.

The cosmopolitan genotype sequences of DENV-2 are classified into lineage 5. These lineage classifications were picked up by Yenamandra *et al*. ^31^, who highlighted the great heterogeneity of the cosmopolitan genotype and the distribution of genotypes over time. This suggests that the cosmopolitan genotype varies genetically over time and gives way to several lineages circulating in different countries.

Our findings suggest circulation of the Asian American genotype in 2019 and 2020, and its subsequent replacement by the cosmopolitan genotype that emerged in 2019.

This study presents important results, but also some limitations that may affect the interpretation of these results. Although samples were collected from several regions of Peru, they do not cover all regions of the country, which could bias the representation of dengue genotypes. The study mainly analyzes genetic information of Dengue viruses, without integrating clinical-epidemiological factors, which restricts the evaluation of the impact of genotypes on the severity of the disease. In addition, an analysis that integrates the study of specific ecological and environmental factors limits could improve the knowledge of a more realistic situation of Dengue disease in Peru.

In conclusion, our findings demonstrate the rapid dispersal and co-circulation of DENV-1 and DENV-2 genotypes in different regions of Peru during 2019-2022. These results highlight the urgent need to strengthen epidemiological surveillance systems and genomic studies for the timely detection of emerging DENV genotypes and their potential spread to other countries in the Americas.

## References

[1] Pan American Health Organization, World Organization of Health (2023). Epidemiological Alert: Increase in dengue cases in Central America and the Caribbean. 15 September 2023.

[2] Cabezas C, Fiestas V, García-Mendoza M, Palomino M, Mamani E, Donaires F (2015). Dengue in Peru: a quarter century after its reemergence. Rev Peru Med Exp Salud Publica.

[3] Pan American Health Organization, World Organization of Health (2023). Epidemiological Update for Dengue, Chikungunya and Zika in 2022. 25 June 2023.

[4] Sala situacional de Dengue.

[5] García MP, Padilla C, Figueroa D, Manrique C, Cabezas C (2022). Emergence of the Cosmopolitan genotype of dengue virus serotype 2 (DENV2) in Madre de Dios, Peru, 2019. Rev Peru Med Exp Salud Publica.

[6] Carrasco J, Cabrera P, Sampen G, Díaz-Vélez C Perfil clínico, epidemiológico y geográfico de casos de dengue durante el fenómeno El Niño Costero 2017, Lambayeque-Perú. Rev Haban Cienc Med.

[7] Amorim M, Hernández L, Naveca F (2023). Emergence of a New Strain of DENV-2 in South America Introduction of the Cosmopolitan Genotype through the Brazilian-Peruvian Border. Trop Med Infect Dis.

[8] Domingo C, Palacios G, Jabado O, Reyes N, Niedrig M, Gascón J (2006). Use of a Short Fragment of the C-Terminal E Gene for Detection and Characterization of Two New Lineages of Dengue Virus 1 in India. J Clin Microbiol.

[9] Suchard MA, Lemey P, Baele G, Ayres DL, Drummond AJ, Rambaut A (2018). Bayesian phylogenetic and phylodynamic data integration using BEAST 1 10. Virus Evol.

[10] Rambaut A, Lam TT, Max Carvalho L, Pybus OG (2016). Exploring the temporal structure of heterochronous sequences using TempEst (formerly Path-O-Gen). Virus Evol.

[11] Darriba D, Taboada GL, Doallo R, Posada D (2012). jModelTest 2: more models, new heuristics and parallel computing. Nat Methods.

[12] Lemey P, Rambaut A, Drummond AJ, Suchard MA (2009). Bayesian phylogeography finds its roots. PLoS Comput Biol.

[13] Rambaut A, Drummond AJ, Xie D, Baele G, Suchard MA (2018). Posterior Summarization in Bayesian Phylogenetics Using Tracer 1 7. Syst Biol.

[14] Faria NR, Nogueira RM, de Filippis AM (2013). Twenty years of DENV-2 activity in Brazil molecular characterization and phylogeny of strains isolated from 1990 to 2010. PLoS Negl Trop Dis.

[15] Cabezas C (2005). Dengue en el Perú: Aportes para su diagnóstico y control. Rev Peru Med Exp Salud Publica.

[16] Dostal T, Meisner J, Munayco C, García PJ, Cárcamo C, Pérez-Lu JE (2022). The effect of weather and climate on dengue outbreak risk in Peru, 2000-2018: A time-series analysis. PLoS Negl Trop Dis.

[17] Morrison A, Minnick S, Rocha C, Forshey BM, Stoddard ST, Getis A (2010). Epidemiology of dengue virus in Iquitos, Peru 1999 to 2005: interepidemic and epidemic patterns of transmission. PLoS Negl Trop Dis.

[18] Mamani E, Álvarez C, García M M, Figueroa D, Gatti M, Guio H (2011). Circulación de un linaje diferente del virus dengue 2 genotipo América / Asia en la región amazónica de Perú, 2010. Rev Peru Med Exp Salud Publica.

[19] Williams M, Mayer SV, Johnson WL, Chen R, Volkova E, Vilcarromero S (2014). Lineage II of Southeast Asian/American DENV-2 Is Associated with a Severe Dengue Outbreak in the Peruvian Amazon. Am J Trop Med Hyg.

[20] Amorim MT, Hernández LHA, Naveca FG, Essashika Prazeres IT, Wanzeller ALM, Silva EVPD (2023). Emergence of a New Strain of DENV-2 in South America Introduction of the Cosmopolitan Genotype through the Brazilian-Peruvian Border. Trop Med Infect Dis.

[21] Khan E, Prakoso D, Imtiaz K, Malik F, Farooqi JQ, Long MT (2020). The Clinical Features of Co-circulating Dengue Viruses and the Absence of Dengue Hemorrhagic Fever in Pakistan. Front Public Health.

[22] Verma P, Baskey U, Choudhury KR, Dutta S, Bakshi S, Das R (2023). Changing pattern of circulating dengue serotypes in the endemic region An alarming risk to the healthcare system during the pandemic. J Infect Public Health.

[23] Mollinedo Pérez JS, Aymara Mollinedo Z, Gironda WJ, Mollinedo RE, Mollinedo Pérez JS, Aymara Mollinedo Z (2021). Dengue del viajero enfermedades tropicales fuera de los trópicos en Bolivia. Rev Científica Cienc Médica.

[24] Real-Cotto JJ, Regato Arrata ME, Burgos Yépez VE, Jurado Cobeña ET (2017). Evolución del virus dengue en el Ecuador Período 2000 a 2015. An Fac Med.

[25] Gularte JS, Sacchetto L, Demoliner M, Girardi V, da Silva MS, Filippi M (2023). DENV-1 genotype V linked to the 2022 dengue epidemic in Southern Brazil. J Clin Virol.

[26] Ocazionez-Jiménez RE, Ortiz-Báez AS, Gómez-Rangel SY, Miranda-Esquivel DR (2013). Dengue virus serotype 1 (DENV-1) from Colombia its contribution to dengue occurrence in Santander. Biomedica.

[27] Rojas A, Moreira Soares A, Mendoza LP, Acosta ME, Aria L, Páez M (2021). Revisiting the dengue epidemic of 2011 in Paraguay molecular epidemiology of dengue virus in the Asuncion metropolitan area. BMC Infect Dis.

[28] Cruz CD, Forshey BM, Juarez DS, Guevara C, Leguia M, Kochel TJ (2013). Molecular epidemiology of American/Asian genotype DENV-2 in Peru. Infect Genet Evol.

[29] Giovanetti M, Pereira LA, Santiago GA, Fonseca V, Mendoza MPG, de Oliveira C (2022). Emergence of Dengue Virus Serotype 2 Cosmopolitan Genotype, Brazil. Emerg Infect Dis.

[30] Suzuki K, Phadungsombat J, Nakayama EE, Saito A, Egawa A, Sato T (2019). Genotype replacement of dengue virus type 3 and clade replacement of dengue virus type 2 genotype Cosmopolitan in Dhaka, Bangladesh in 2017. Infect Genet Evol.

[31] Yenamandra SP, Koo C, Chiang S, Lim HSJ, Yeo ZY, Ng LC (2021). Evolution, heterogeneity and global dispersal of cosmopolitan genotype of Dengue virus type 2. Sci Rep.

