# Supplementary material for: Rapid spread of the emerging cosmopolitan genotype of dengue virus serotype 2, and expansion of dengue virus serotype 1 genotype V in Peru
Source: Rev Peru Med Exp Salud Publica. 2024 Dec 2;41(4):375–84. doi: 10.17843/rpmesp.2024.414.13898 (PMC11797585; doi:10.17843/rpmesp.2024.414.13898)
Supplement: Supplementary material. — Available in the electronic version of the RPMESP. [file rpmesp-41-04-13898-s001.zip › rpmesp-41-04-13898.docx]

**MATERIAL SUPLEMENTARIO**

**A**

**
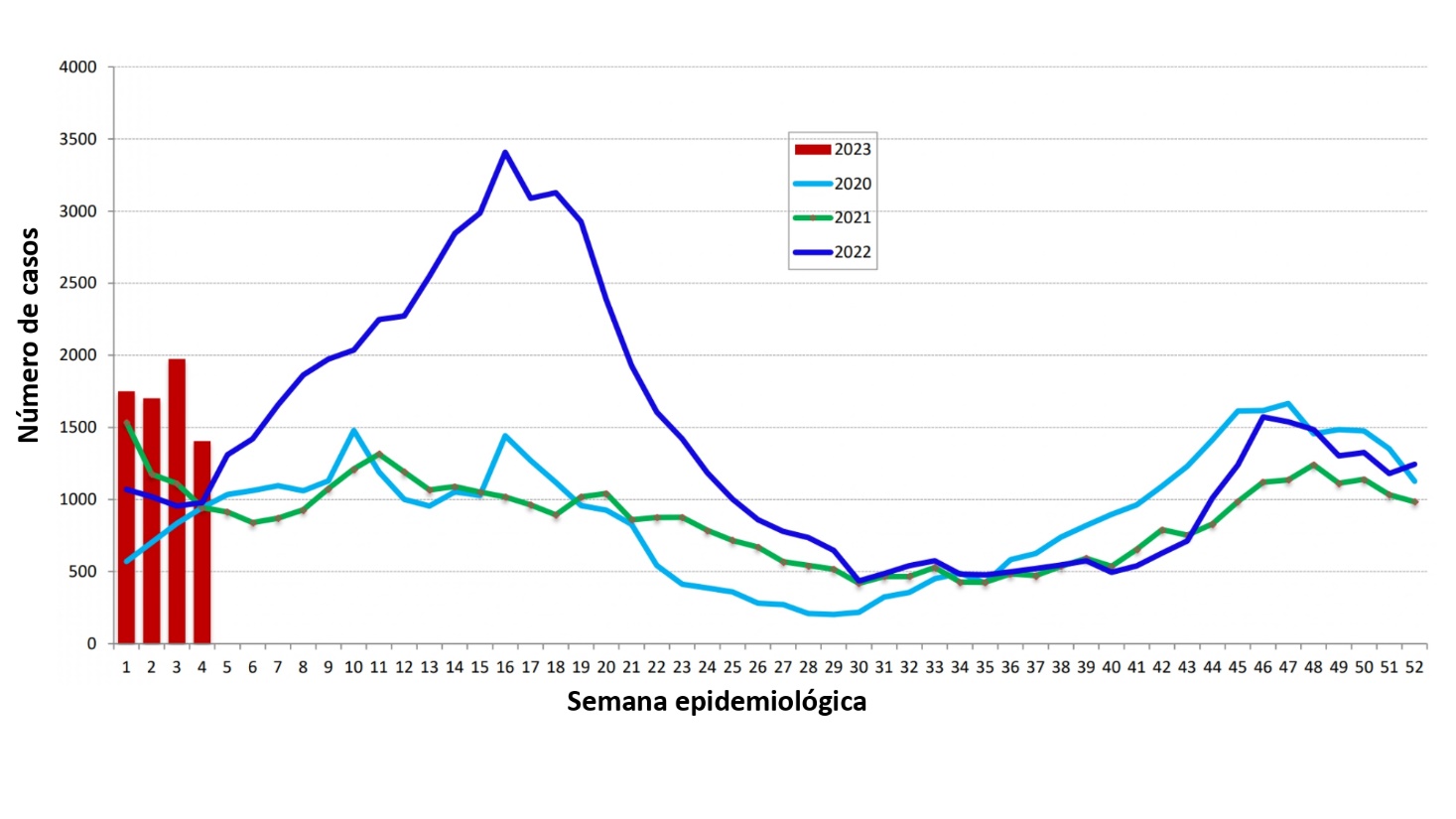
**

**B**


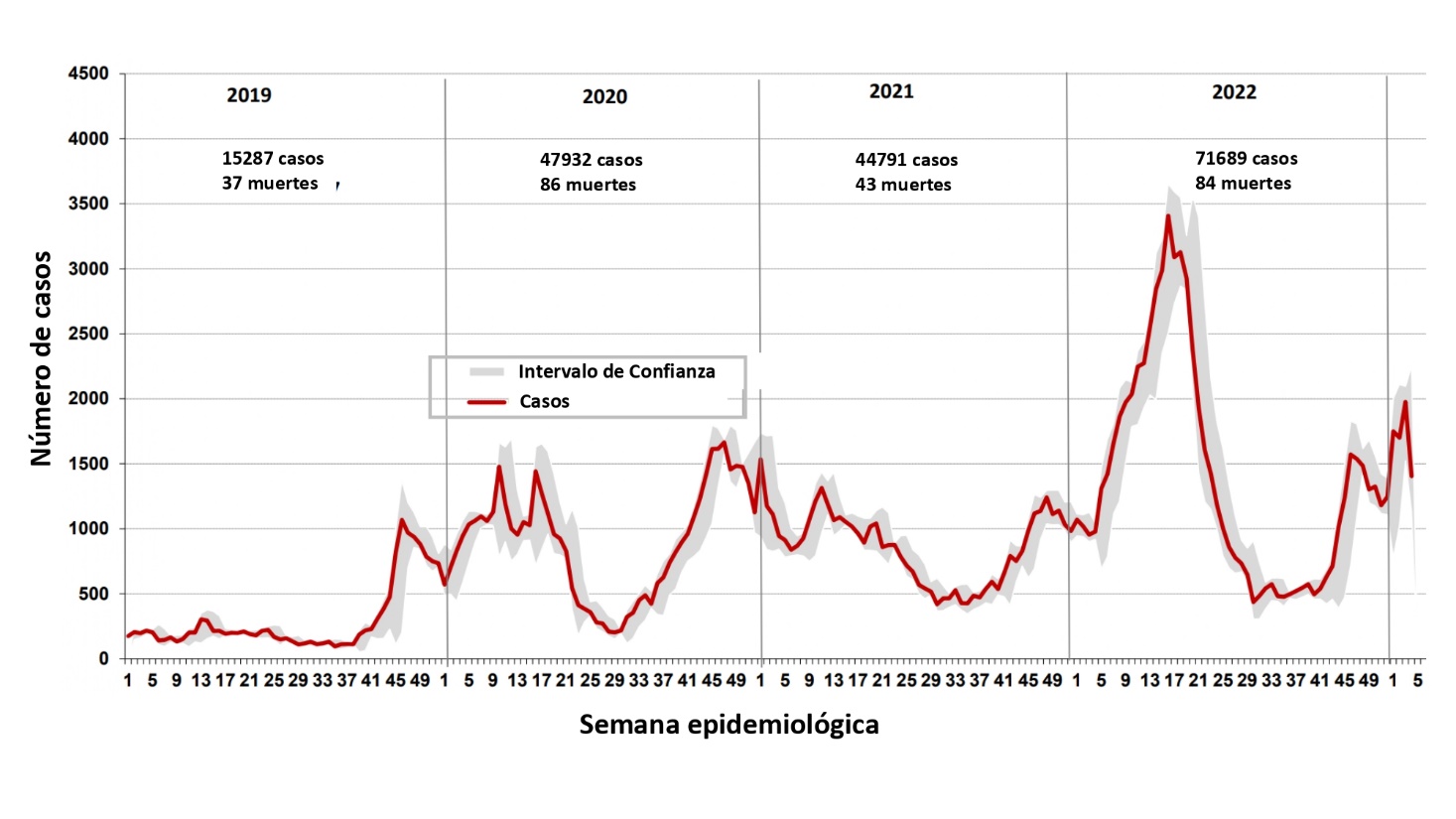


**Figura S1. Tendencias de notificación y reporte de casos de Dengue en Perú del 2019 al 2022.** La imagen fue tomada del sitio web del Centro Nacional de Epidemiología, Prevención y Control de Enfermedades del Perú. El panel A compara el número de casos notificados de la enfermedad del Dengue de 2020 a 2022. El panel B muestra las tendencias en la notificación de la enfermedad del Dengue en Perú de 2019 a 2022.


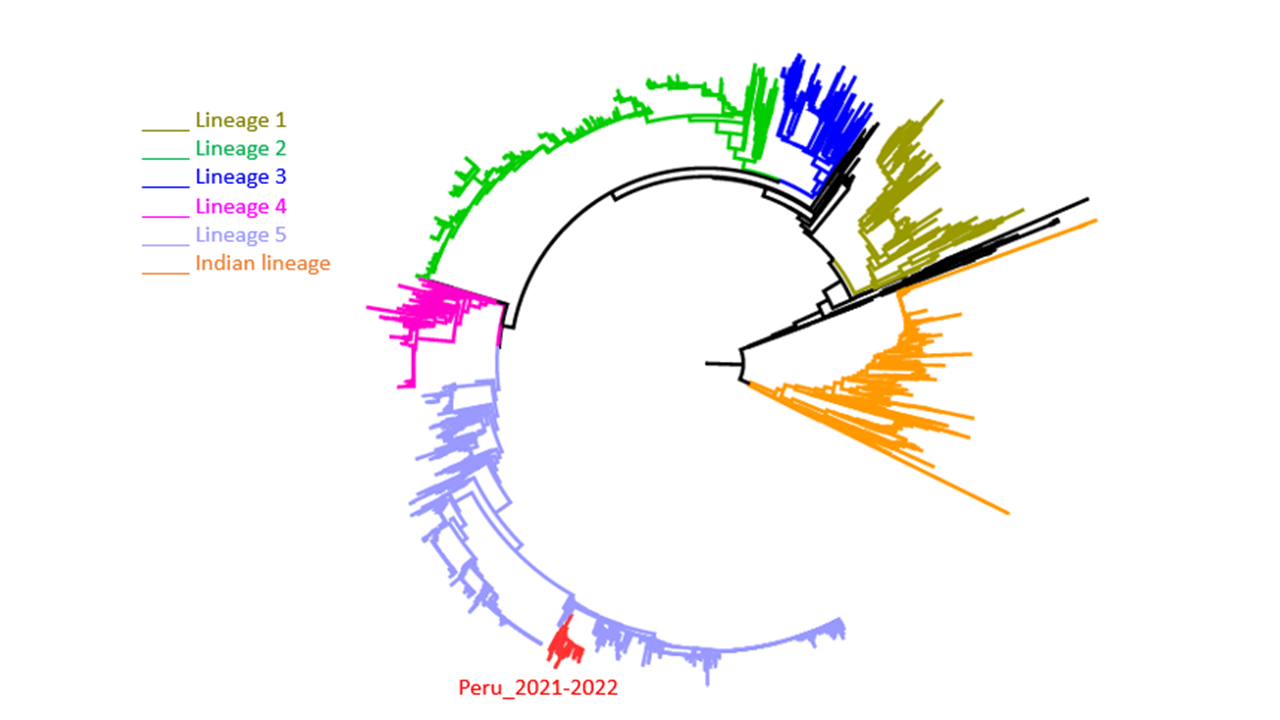


**Figura S2. Clasificación de linajes del genotipo cosmopolita del DENV-2. Las secuencias peruanas resaltadas en rojo pertenecen al linaje 5, descrito previamente [32].** Las secuencias peruanas, resaltadas en rojo, pertenecen al linaje 5, como se describió previamente [32]. El análisis incluyó 3.827 secuencias completas del gen E del genotipo cosmopolita del DENV-2, incluidas las secuencias del estudio y las recuperadas de la base de datos del NCBI.

**
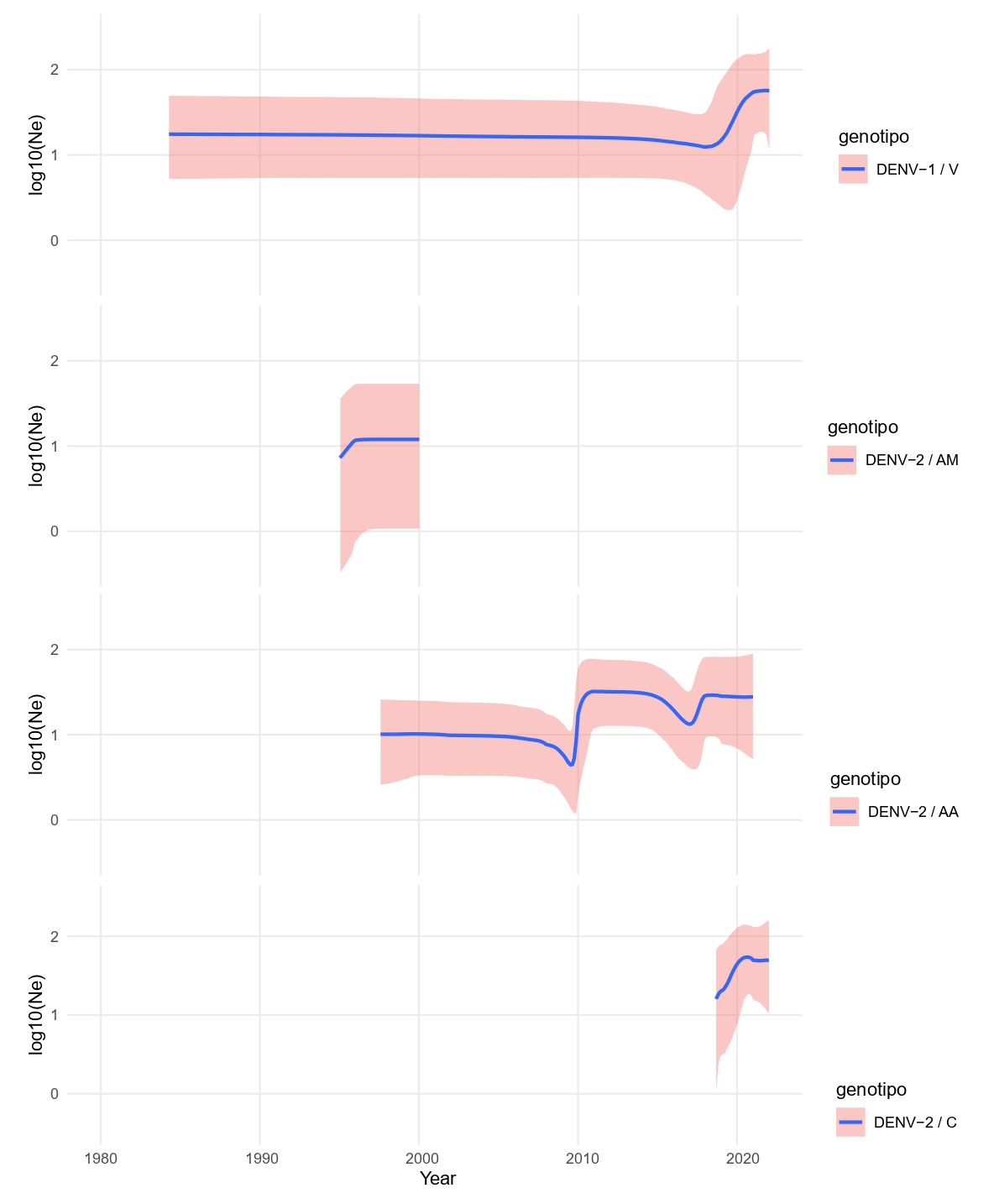
**

**Figura S3.** Skyline Plots bayesianos que representan la variación temporal del tamaño efectivo de la población (Ne) en escala logarítmica para los genotipos DENV1 (genotipo V) y DENV2 (americano, am; asiático-americano, aa; cosmopolita, c). El genotipo América-Asia de DENV-2 presentó dos linajes, el linaje I del 2001 al 2019, y el linaje II del 2009 en adelante (29).
