# Supplementary material for: Rapid spread of the emerging cosmopolitan genotype of dengue virus serotype 2, and expansion of dengue virus serotype 1 genotype V in Peru
Source: Rev Peru Med Exp Salud Publica. 2024 Dec 2;41(4):375–84. doi: 10.17843/rpmesp.2024.414.13898 (PMC11797585; doi:10.17843/rpmesp.2024.414.13898)
Supplement: Translation

# Translation: Rápida propagación del genotipo emergente cosmopolita del virus dengue serotipo 2, y expansión del genotipo V de dengue serotipo 1 en el Perú

**Authors:** Henri Bailon, Víctor Jimenez, Marco Galarza, Princesa Medrano, Orson Mestanza, Dana Figueroa, Wendy Lizarraga, Iris Silva, Luren Sevilla, Verónica Hurtado, Vanessa Izarra, Carlos Padilla, Luis Barcena, Omar Caceres, Susy Merino, Adolfo Marcelo, Nora Ruiz, Hapuarachchige Chanditha Hapuarachchii, César Cabezas Sánchez, María P. García

**Affiliations:** 1 Área de Innovación y Desarrollo (Equipo de Vigilancia Genómica), Centro Nacional de Salud Pública (CNSP), Instituto Nacional de Salud (INS), Lima, Perú.; 2 Laboratorio Nacional de Referencia de Enfermedades Metaxénicas y Zoonosis Virales, CNSP-INS, Lima, Perú.; 3 Laboratorio Nacional de Referencia de Virus Inmunoprevenibles, CNSP-INS, Lima, Perú.; 4 División de Microbiología y Epidemiología Molecular, Instituto de Salud Medioambiental, Agencia Nacional de Medio Ambiente, Singapur.; 5 Instituto Nacional de Salud, Lima, Perú.

**Keywords:** Perú, Virus del Dengue, Genotipo, Serotipo, DENV-1, DENV-2

## Abstract

**Objetivos:**

. En el presente estudio se evaluó la prevalencia y distribución del genotipo V del virus del dengue serotipo 1 (DENV-1) y del genotipo cosmopolita serotipo 2 (DENV-2) en Perú entre los años 2019 y 2022.

**Materiales y métodos.:**

La región del gen de la envoltura (E) se amplificó a partir de 79 muestras de suero mediante reacción en cadena de la polimerasa (PCR) y se secuenció mediante tecnología de secuenciación de nueva generación (NGS). Las secuencias obtenidas se analizaron posteriormente mediante herramientas bioinformáticas.

**Resultados.:**

El estudio generó secuencias del gen de la envoltura de los serotipos DENV-1 y DENV-2. Nuestro estudio reveló una rápida dispersión y amplia distribución del genotipo cosmopolita DENV-2 en diversas regiones del Perú en 2022, así como la propagación del genotipo V DENV-1 a nuevas regiones peruanas, junto con el genotipo cosmopolita DENV-2.

**Conclusiones.:**

Nuestros hallazgos sugieren la urgente necesidad de fortalecer los sistemas de vigilancia epidemiológica y genómica para conocer y controlar la expansión de los genotipos circulantes del DENV en Perú. Esto permitirá una respuesta más rápida, a la vez que el monitoreo de su potencial diseminación a otros países de las Américas.

## INTRODUCCIÓN

El virus del dengue (DENV) es un virus ARN perteneciente a la familia Flaviviridae e incluye cuatro serotipos, DENV-1, DENV-2, DENV-3 y DENV-4. El DENV causa una enfermedad febril (fiebre del dengue) y es clasificada como sin signo de alarma y grave, y en ciertos casos puede ser mortal. La infección por un serotipo concreto del DENV sólo provoca inmunidad de por vida contra ese serotipo específico, lo que deja el riesgo de reinfección por otro serotipo. Se sabe que estas infecciones secundarias por DENV son más graves que las infecciones primarias ^1^.

Los países de América Central y del Sur, así como los del Caribe, registran anualmente una elevada carga de dengue, que ha ido en aumento a lo largo de los años.

El dengue es de gran importancia para la salud pública desde que se notificó en Perú en la década de 1990 ^2^. La incidencia del dengue en Perú ha aumentado a lo largo de los años debido a la presencia de *Aedes aegypti,* el principal vector del DENV. Los cuatro serotipos del DENV han estado presentes en Perú desde su aparición. Sin embargo, la ocurrencia y prevalencia de serotipos específicos y sus genotipos asociados han variado con el tiempo.

Perú tiene la segunda carga más alta de dengue en las Américas, después de Brasil. En 2022, se notificaron 47,656 casos, de los cuales el 87,4% fue dengue sin signos de alarma, el 12,3% fue dengue con signos de alarma y el 0,3% fue dengue grave, lo que provocó un total de 65 muertes. En comparación, el país notificó 21.407 casos con una tasa de incidencia de 64,17 casos por 100.000 habitantes durante el mismo período en 2021 ^3^. El mayor número de casos notificados en 2022 se produjo en las regiones peruanas de Loreto, Ica, Ucayali, Cusco, Cajamarca, San Martín, Lambayeque, Madre de Dios, Huánuco, Ancash y Junín ^4^.

La ecología, el clima y la biodiversidad de Perú proporcionan un entorno propicio para la reproducción del *Aedes aegypti*. La omnipresencia de tales hábitats ha propiciado una mayor dispersión del vector. Entre los cuatro serotipos del DENV, el DENV-1 (en particular el genotipo V) ha sido el más frecuentemente notificado en varios departamentos de Perú, con casos notificados en Iquitos desde la década de 1990, y el DENV-2 (genotipo americano) se introdujo en Perú posteriormente. El genotipo americano/asiático del DENV-2 (linaje II) emergió a finales del 2010 en Perú, causando muchos brotes y una epidemia por dengue a nivel nacional ^4^. El genotipo cosmopolita del DENV-2 se reportó por primera vez en el Perú durante un brote en 2019 ^5^. Posteriormente se propagó a diferentes departamentos, incluidos Piura, Lambayeque, Ica, Madre de Dios, Ancash, Cajamarca, San Martín, Loreto, Huánuco y Ucayali, en 2022 ^6^. En 2021, Brasil también notificó un caso infectado con el genotipo cosmopolita del DENV-2, lo que indica su probable propagación a otros países vecinos ^7^. En consecuencia, se aconsejó a los países de la región que intensifiquen sus esfuerzos de vigilancia genómica.

Por lo tanto, es crucial contar con una vigilancia eficaz del DENV en Perú para monitorear la propagación de nuevos genotipos circulantes, como el genotipo cosmopolita. Esto se debe a que su rápida dispersión geográfica se ha relacionado con un aumento en el número de casos de dengue. El presente estudio tuvo como objetivo identificar la prevalencia y distribución del genotipo V del DENV-1 y del genotipo cosmopolita del DENV-2 en Perú utilizando tecnología de secuenciación de próxima generación (NGS) y análisis filogenéticos basados en el gen de la envoltura. En este estudio, revelamos una expansión del genotipo V del DENV-1 preexistente en Perú y una rápida propagación del genotipo cosmopolita del DENV-2 en 2022 a diferentes regiones de Perú, tras su introducción en 2019.

**MENSAJE CLAVE**

Motivación para realizar el estudio. El estudio busca analizar la prevalencia y distribución de los genotipos V del virus del dengue tipo 1 (DENV-1) y del genotipo cosmopolita DENV-2 en Perú, con el fin de entender su expansión en diferentes regiones; ya que estos virus pueden ocasionar importantes brotes en el país.

Principales hallazgos. Se observó una rápida dispersión y amplia distribución del genotipo cosmopolita DENV-2 en Perú en 2022, tras su introducción inicial en 2019. El genotipo V de DENV-1, presente en Perú desde la década de 1990, actualmente se ha expandido a nuevas regiones peruanas, luego de estar restringido anteriormente a zonas rurales y de selva en el norte del país.

Implicancias. Los resultados resaltan la urgente necesidad de reforzar los sistemas de vigilancia epidemiológica y genómica para monitorear y controlar la propagación de estos genotipos en Perú, permitiendo una respuesta más rápida y controlando su potencial diseminación a otras regiones de las Américas.

## MATERIALES Y MÉTODOS

### Datos de la muestra

El estudio analizó 79 muestras positivas para dengue, recogidas en Perú por el Instituto Nacional de Salud como parte del diagnóstico y vigilancia de los arbovirus de las enfermedades metaxénicas virales. Además, el Centro de Control y Prevención de Enfermedades (CDC) de Perú proporcionó información epidemiológica sobre los serotipos circulantes de DENV de 2019 a 2022.

### Extracción de material genético y secuenciación

El ARN viral se extrajo del suero humano utilizando el kit de purificación de ADN/ARN viral MagaBio plus III (BioFlux) siguiendo el protocolo recomendado por el fabricante. Posteriormente, el gen de la envoltura del DENV (gen *E*) se amplificó mediante la tecnología de reacción en cadena de la polimerasa con trasncripción reversa (RT-PCR) utilizando el kit Superscript III Platinum one-step RT-PCR (Invitrogen™) y los cebadores o primers específicos. Los productos amplificados se purificaron con el kit Mag-Bind Total Pure NGS (Omega Bio-Tek) y se secuenciaron con el kit Nextera XT (Illumina) en el secuenciador genómico MiSeq (Illumina).

### Procesamiento de datos de secuencias y análisis filogenético

La calidad de lectura de las secuencias se evaluó mediante la herramienta FASTQC v.0.11.9 (https://www.bioinformatics.babraham.ac.uk/projects/fastqc/). A continuación, las secuencias de buena calidad (valor de calidad Q superior a 20) se ensamblaron con la secuencia del gen E de la referencia del NCBI de DENV-1 (NC_001477.1) y DENV-2 (NC_001474.2) utilizando los programas BWA v.0.0.17, samtools v.1.14 e IVAR v.1.3.1. El editor Aliview v.1.28 y el visor integrador de genómica (IGV) v.2.16.0 se utilizaron para curar manualmente las secuencias consenso.

Para el análisis filogenético, se recuperaron las secuencias completas del gen *E* del DENV-1^8^ (n=1.338) y del DENV-2 (n=1.521) de la base de datos de virus del NCBI. Además, también se incluyeron 14 muestras positivas de DENV-2 recogidas durante los brotes de dengue de diciembre de 2019 y enero de 2020 en Perú. Se utilizó el programa MAFFT v7.475 para realizar el alineamiento múltiple de secuencias. Para realizar los análisis filogenéticos se utilizó el método de Máxima Verosimilitud (ML), implementado en el programa RAxML v8.2.10. El árbol ML resultante se visualizó con Microreact (https://microreact.org/).

### Análisis bayesianos

*Filogenia bayesiana*

Se construyó una filogenia bayesiana a escala temporal para el DENV-2 utilizando el paquete de software Bayesian Evolutionary Analysis by Sampling Trees (BEAST) v1.10.4 ^9^. El conjunto de datos consistió en 202 secuencias completas del gen *E del* DENV-2, incluyendo 125 secuencias peruanas y 77 secuencias globales recuperadas de la base de datos del NCBI. La señal temporal del conjunto de datos final para cada serotipo se comprobó utilizando TempEst versión 1.5.3 ^10^. Se utilizó el programa jModelTest ^11^ para determinar que el modelo de sustitución General Time Reversible (GTR+G4+I) era el que mejor se ajustaba. Para evitar asumir cualquier escenario demográfico particular, se utilizó un reloj relajado log normal no correlacionado y el previo Análisis bayesiano coalescente (*Bayesian Coalescent Skyline Plot*) (10 pasos) para construir el árbol escalado en el tiempo. El análisis Markov Chain Monte Carlo (MCMC) ^12^ se ejecutó durante 100 millones de generaciones, con una muestra tomada cada 10.000 estados. Los archivos de registro de salida se visualizaron con Tracer v.1.5 ^13^. Se consideró que un tamaño de muestreo efectivo (ESS) >200 era suficiente para la convergencia de los parámetros. El árbol de máxima credibilidad cladística (MCC) se construyó tras eliminar el primer 10% de todos los árboles (burn-in) utilizando TreeAnnotator v.1.7.4. El árbol MCC fue graficado en FigTree v.1.4.3 (http://tree.bio.ed.ac.uk/software/figtree/).

*Estimaciones del tamaño efectivo de la población*

La variación temporal del tamaño efectivo de la población (EPS) y los tiempos de divergencia se determinaron mediante el método bayesiano implementado en el software BEAST v1.10.4. Se realizaron dos ejecuciones MCMC independientes, cada una de 50 millones de generaciones con muestreos cada 5000 generaciones. Se utilizaron los parámetros a priori por defecto. El análisis bayesiano se calibró en el tiempo e incluyó el modelo skyline coalescent, así como los modelos de reloj molecular estricto y no correlacionado, y el modelo de sustitución GTR+I+G4. Para determinar la convergencia estadísticamente robusta, se utilizó TRACER v1.7.1 (http://tree.bio.ed.ac.uk/software/tracer/), considerándose robustos los valores de ESS superiores a 200. Los datos restantes se combinaron en Log Combiner v1.10 tras un periodo de combustión del 10%. Se generó un árbol resumen con calibración temporal utilizando TreeAnnotator v.1.10 (https://beast.community/treeannotator) y se visualizó en FigTree v1.4.

*Clasificación de linajes del genotipo cosmopolita DENV-2*

La clasificación por linajes del genotipo cosmopolita se llevó a cabo construyendo un árbol filogenético Neighbour Joining utilizando el modelo de sustitución de parámetros Kimura-2 con parámetro gamma y sitios invariantes (Г5 + I) en el paquete de software MEGA 7 (https://academic.oup.com/mbe/article/33/7/1870/2579089). El análisis comprendió 3.827 secuencias completas del gen *E* del genotipo cosmopolita DENV-2, incluidas las secuencias del estudio y las obtenidas de la base de datos del NCBI. El árbol se visualizó y anotó con FigTree v.1.4.3 (http://tree.bio.ed.ac.uk/software/figtree/).

### Cuestiones éticas

El estudio no fue aprobado por un comité de ética porque los datos proceden de la actividad de vigilancia del laboratorio y no de una investigación específica. No obstante, los datos se han tratado de acuerdo con las normas y políticas de tratamiento de datos de la institución.

## RESULTADOS

Los casos de dengue suelen aumentar en Perú durante las estaciones de primavera y verano de cada año. Según los datos del Centro Peruano de Control y Prevención de Enfermedades, el mayor número de casos de dengue se produjo entre las semanas 14 y 18 de 2022 (material suplementario S1). Además, los gráficos acumulados muestran un aumento significativo de los casos de dengue a partir de 2019 (material suplementario S1).

Perú solo ha detectado DENV-1 (genotipo V) y DENV-2 (genotipos asiático-americano y cosmopolita) entre los cuatro serotipos de DENV desde 2019 (Tabla 1). La distribución geográfica y la composición por serotipos de las 79 muestras positivas para DENV analizadas en este estudio se resumen en la Tabla 2.

**Tabla 1:** Introducción y circulación en distintas épocas de los serotipos/genotipos del DENV en el Perú.

Serotipo	Año de ingreso al Perú	Genotipos circulantes hasta el 2019	Genotipos circulantes hasta el 2022
DENV-1	1990	Genotipo III	
		Genotipo V	Genotipo V
DENV-2	2001/2010	América/Asia	
		América	
	2019	Cosmopolita	Cosmopolita
DENV-3	2001	India-Genotipo III	
DENV-4	2001	Indonesia-Genotipo II	

**Tabla 2:** Distribución de los 79 casos positivos de dengue incluidos en este estudio.

Departamento	Región	DENV1/V		DENV2/C	
		2021	2022	2021	2022
Piura	Costa	-	1	-	-
Lambayeque		-	2	-	1
Ancash		-	3	-	-
La Libertad		-	-	2	-
Lima		-	5	3	7
Ica		1	1	-	-
Cajamarca	Sierra	-	3	2	3
Huánuco		-	-	2	3
Junín		1	2	2	3
Pasco		-	-	1	-
Ayacucho		-	-	2	2
Puno		1	-	-	-
Cusco		-	1	1	3
Loreto	Selva	-	3	1	2
San Martin		1	-	1	-
Ucayali		2	2	-	2
Madre de Dios		-	-	2	1
Amazonas		-	-	4	

DENV1-V: Virus del dengue serotipo 1, genotipo V

DENV2-C: Virus del dengue serotipo 2, genotipo cosmopolita

Los resultados de este estudio mostraron que el genotipo cosmopolita DENV-2 fue el más frecuente entre las muestras de estudio, representando el 63,3% de las muestras recogidas en las diferentes regiones de Perú. El genotipo cosmopolita DENV-2 causó el mayor número de casos en Lima, pero también se observó en otras regiones como Amazonas, Ayacucho, Cajamarca, Cusco, Huánuco, Junín, Loreto, Lambayeque, La Libertad, Madre de Dios, Pasco y Ucayali. Adicionalmente, el genotipo V del DENV-1 co-circuló en las regiones de Lima, Loreto, Cuzco, Ucayali, San Martín, Cajamarca y Lambayeque (Tabla 2 y figura 1).

**Figura 1:** Frecuencia y distribución de infecciones por Dengue en el Perú por serotipo y genotipo del 2018 al 2022. El panel A muestra la frecuencia de infecciones por Dengue en Perú por serotipo y genotipo en 2022. La distribución de la frecuencia de cada serotipo está representada por un color dife-rente. El panel B muestra la distribución geográfica de DENV-1 (genotipo V) y DENV-2 (genotipo cosmopolita) en Perú durante el periodo 2018-2022.

En 2021, hubo más casos del genotipo cosmopolita DENV-2 que del genotipo V DENV-1 en las regiones de selva y montaña. Las regiones costeras notificaron pocos o ningún caso de estos dos genotipos. En 2022, ambos genotipos mostraron una mayor dispersión, con el genotipo cosmopolita DENV-2 expandiéndose más en las regiones de selva y montaña, y el genotipo V DENV-1 expandiéndose en las regiones costeras (Tabla 2). La carga de dengue en el Perú en 2022 fue impulsada tanto por el genotipo V del DENV-1 como por el genotipo cosmopolita del DENV-2, que se expandieron rápidamente en las regiones costeras peruanas en 2021 y 2022.

Las mutaciones no sinónimas en el gen *E* del genotipo cosmopolita DENV-2 incluyen: M6I; V15I; Q52H; E71A; F119L; R120T; V129I; V140A; H149N; T180I; N203S; P222S; T226I; S255P; V309A; D329E; I380V; N390S; V428M; F429L; I462V; T478S e I484V, siendo todas las mutaciones resaltadas en negrita comunes a todas las secuencias (Material suplementario: Cod. de acceso GISAID y mutaciones DENV gen E).

Las secuencias del genotipo V del DENV-1 están genéticamente relacionadas con las de países de la región, como Ecuador, Colombia, Venezuela y México. Se observaron dos clados distintos dentro del genotipo V del DENV-1 de Perú. El clado dominante incluía muestras de casi todas las regiones peruanas, en particular de las regiones norte y centro, y estaba relacionado con secuencias de Ecuador (Figura 2A). El segundo cluster comprendía muestras de unas pocas regiones del sur del Perú (Figura 2B). La detección inicial del cluster dominante en Lima e Ica en 2021, antes de su aparición en otras regiones en 2022, sugirió una posible ruta de dispersión desde la costa central hacia las regiones del norte y sur del Perú. Del mismo modo, el cluster menos dominante se detectó por primera vez en la región central de Junín en 2021, antes de emerger en Cusco, Ancash, Ucayali y Puno (Figura 2B).

**Figura 2:** Árbol filogenético de máxima verosimilitud del DENV-1. El panel 3A muestra la filogenia global con las muestras peruanas resalta-das en barras azules y los genotipos mostrados en diferentes colores. El panel 3B muestra un subárbol filogenético detallado con las muestras peruanas representadas por círculos azules. Los cambios estimados por sitio están representados por escalas de barras horizontales.

El genotipo V del DENV-1 presentaba las siguientes sustituciones de aminoácidos en el gen *E* : D317N; N52D; T88A; I114L; T161I; K203E; I293T; M297V; S338L; S339T; A369T; V345L; T368A; I394L; I436V; T441I; K483E; I439V; I457V; I573T; S619T; A649T; I716V; I719V; V482I y M484I, con las mutaciones resaltadas en negrita, siendo comunes en todas las muestras analizadas (Material suplementario S2).

Desde la primera aparición del DENV-2 en Perú, se han identificado tres genotipos: Americano, Asiático-Americano (AA) y Cosmopolita. El genotipo cosmopolita del DENV-2 que ha surgido recientemente en Perú está genéticamente relacionado con los notificados anteriormente en Bangladesh ^5^. Los primeros casos del genotipo cosmopolita DENV-2 en Perú fueron reportados en diciembre de 2019 en las regiones de Madre de Dios, Puno y Cusco ^5^. Estos casos también estaban genéticamente relacionados con los encontrados en Bangladesh. En 2021, se notificaron casos en Madre de Dios, Amazonas, Junín, Cajamarca, Pasco, Ucayali, Loreto, Cusco, Ayacucho, Huánuco y San Martín. El genotipo cosmopolita fue el único genotipo circulante del DENV-2 en todas las regiones del país en 2022 (Figura 3B).

**Figura 3:** Árbol filogenético de máxima verosimilitud del DENV-2. El panel A muestra la filogenia global con las muestras peruanas resalta-das en barras naranjas y los genotipos mostrados en diferentes colores. El panel B muestra un subárbol filogenético detallado con las muestras peruanas en círculos naranjas. Los cambios estimados por sitio están representados por escalas de barras horizontales.

Los hallazgos de nuestro estudio indicaron que el genotipo cosmopolita fue dominante después de 2019 para el serotipo 2; ya que el mayor número de casos de dengue fue causado por el genotipo cosmopolita en todas las regiones del Perú donde se presentaron casos en 2021 y 2022 (Figura 4).

**Figura 4:** Dominancia de los genotipos de DENV-2 en Perú antes y después de 2019. El árbol escalado en el tiempo se construyó utilizando el paquete de software BEAST 1.10.4, empleando un modelo general de sustitución reversible en el tiempo (GTR+G4+I), un modelo de reloj relajado no correlacionado lognormal, y un árbol skyline bayesiano a priori. La longitud de la cadena Markov Chain Monte Carlo fue de 100 millones, con árboles muestreados cada 10.000 iteraciones. Los análisis incluyeron 202 secuencias completas del gen de la envoltura.

Nuestro estudio reveló que el linaje 5 del genotipo cosmopolita fue el linaje DENV-2 dominante en 2021- 2022. Este hallazgo coincide con un reporte en Brasil en noviembre del año 2021^14^.

El análisis mediante el modelo Bayesiano de la línea del horizonte indicó un tamaño efectivo medio estable de la población del genotipo V del DENV-1 a largo plazo, con un ligero aumento antes de 2020. Por otro lado, el tamaño efectivo de la población del genotipo cosmopolita DENV-2 mostró un rápido aumento tras su aparición en Perú en 2019 (material suplementario S3).

## DISCUSIÓN

Este estudio evidencia la rápida propagación del genotipo cosmopolita del virus del dengue serotipo 2 (DENV-2) y la expansión del genotipo V del serotipo 1 (DENV-1) en Perú entre 2019 y 2022. Desde su introducción en 2019, el genotipo cosmopolita de DENV-2 ha predominado en diversas regiones, extendiéndose rápidamente y reemplazando genotipos previos como el asiático americano. Asimismo, la circulación del DENV-1 genotipo V, presente desde la década de 1990 en zonas selváticas y rurales, también mostró un patrón de expansión hacia nuevas áreas. Estos hallazgos resaltan la necesidad urgente de fortalecer la vigilancia epidemiológica y genómica para controlar la diseminación de estos genotipos y evaluar su potencial de propagación en las Américas.

El dengue es endémico en Perú desde hace varios años y afecta a casi todas las regiones del país. La transmisión del DENV se ve favorecida por diversos factores, como la temperatura, las precipitaciones, la distribución de los mosquitos vectores, los depósitos de agua y las malas condiciones de vivienda ^15^. Las diferentes regiones del Perú albergan una gran variedad de mosquitos; siendo la especie más prevalente *Aedes aegypti*, así como algunas especies de los géneros *Anopheles* y *Culex*, que se encuentran principalmente en las regiones boscosas ^16^.

El serotipo 1 del virus del dengue se identificó por primera vez en 1990, y sus genotipos III y V han estado circulando desde 2019 hasta 2022 ^17^. El 2010 emergió el DENV 2 genotipo asiático americano linaje II y se dispersó rápidamente por muchas ciudades amazónicas y otras regiones del Perú, desplazando al serotipo predominante en esas áreas amazónicas que era DENV-4; desplazando además al DENV -2 genotipo americano asiático linaje I, pero que presentaba pocos casos ^18^^,^^19^.

A pesar de su gran importancia para la salud pública, el dengue ha recibido menos prioridad desde 2019 debido a la gran atención y recursos dedicados al COVID-19. Esto coincidió con la aparición y amplia dispersión del genotipo cosmopolita DENV-2, junto con el genotipo V DENV-1 en Perú. El estudio reveló que el linaje 5 del genotipo cosmopolita ha estado presente en Perú desde 2019. Este linaje también se ha detectado en Brasil, lo que indica su posterior propagación a otros países sudamericanos ^20^.

El análisis bayesiano indicó un tamaño de población efectivo medio estable para el genotipo V del DENV-1 durante un largo periodo de tiempo, en contraste con el rápido aumento del tamaño de la población efectiva del genotipo cosmopolita, tras su aparición en 2019. Se requieren investigaciones adicionales para determinar si la mayor tasa de expansión del genotipo cosmopolita se debe a una mayor ventaja de aptitud en comparación con el genotipo americano/asiático preexistente y el genotipo V de DENV-1. Esto ayudará a determinar el potencial epidémico del genotipo cosmopolita de DENV-2 en Perú. Además de su potencial epidémico, la aparición de un nuevo genotipo también puede dar lugar a cambios en la gravedad de los casos clínicos. En ello pueden influir las comorbilidades, el estado de la infección, la exposición de la población a diferentes serotipos, la variabilidad genética, la edad del paciente, la accesibilidad a los servicios y la experiencia y calidad del manejo clínico de los pacientes ^21^^,^^22^.

Nuestro estudio mostró que el genotipo cosmopolita DENV-2 tuvo una amplia distribución geográfica durante el verano de 2022, lo que indica su rápida propagación desde el primer reporte de unos pocos casos en diciembre de 2019 ^5^.Dada la historia natural de las epidemias de dengue en Perú desde la década de 1990, los resultados resaltan la importancia de mejorar las estrategias de vigilancia y evaluación del riesgo de brotes para guiar medidas efectivas de control de vectores y minimizar la ocurrencia de brotes de dengue. Para determinar la asociación entre la aparición de diferentes genotipos del DENV y la carga de dengue en Perú, es necesario complementar la información clínico-epidemiológica con datos genómicos del virus. La Tabla 2 y la Figura 2 muestran el origen de 79 muestras. De ellas, 29 muestras son de DENV-1 genotipo V reportadas en los departamentos de Ancash, Ica, Piura y Puno. Las 50 muestras restantes son del genotipo cosmopolita DENV-2 y se recogieron en los departamentos de Amazonas, Ayacucho, La Libertad, Madre de Dios y Pasco. En algunos departamentos de Perú se han detectado tanto DENV-1 genotipo V como DENV-2 genotipo cosmopolita. Estos departamentos incluyen Cajamarca, San Martín, Junín, Cusco y Lima. Existen diversos factores que contribuyen a la transmisión del DENV en estas regiones. Los casos de dengue notificados suelen encontrarse en regiones subtropicales y tropicales, aunque también se han dado algunos casos en Puno. Esto puede deberse a la distribución geográfica y/o a los cambios climáticos en las distintas regiones, así como a la presencia del mosquito vector *Aedes aegypti*^6^. Los hallazgos del estudio fueron comparables a los de Mollinedo *et al*. (2021), quienes reportaron que el alto movimiento poblacional de los mosquitos a través de varias zonas ecológicas en climas subtropicales y tropicales es uno de los factores que contribuyen a la mayor incidencia de casos de DENV en Bolivia. El estudio identificó casos de DENV-1 y DENV-2, así como serotipos circulantes de DENV-3 y el primer caso de DENV-4 ^23^.

Real *et al*. ^24^ reportaron un aumento de casos de dengue en Ecuador en los últimos años. El estudio realizado entre 2000-2015 en la provincia de Guayas reveló la presencia de los cuatro serotipos circulantes, con más casos causados por DENV-1 genotipo V y DENV-2 genotipo americano-asiático. En cuanto al DENV-1, los casos peruanos pertenecen al genotipo V, que ha tenido una amplia distribución en diferentes departamentos del Perú. Adicionalmente, se encontró que las secuencias genéticas de varios departamentos del Perú están relacionadas evolutivamente con secuencias de otros países donde se ha reportado DENV-1 genotipo V ^25^.

El análisis filogenético del DENV-1 agrupó las muestras en dos clados distintos. El primer cluster es dominante e incluye muestras de casi todos los departamentos de Perú. El segundo cluster comprende casos pertenecientes al genotipo V del DENV-1 de los años 2021 y 2022, de las regiones sur y norte del Perú. La relación temporal entre los clusters no está clara. Estos resultados son similares a los reportados por Ocasionez R. *et al*. ^26^, quienes encontraron que las cepas en Santander, Colombia pertenecían al genotipo V. Las cepas fueron comparadas con las de países vecinos, como Perú, Venezuela y Brasil, que también mostraron la circulación de otros serotipos.

Rojas *et al*. realizaron un estudio molecular del dengue en Paraguay y descubrieron que la población de Asunción ha experimentado brotes desde 1988, cuando el DENV-1 se introdujo en el país ^27^. Este estudio caracterizó al virus como endémico, y reporta que el serotipo DENV-1 tiene la mayor prevalencia de dengue y co-circula con el DENV-2. Estos resultados coinciden con los de la presente investigación.

En general, las secuencias del genotipo V del DENV-1 de Perú están genéticamente relacionadas con las de Ecuador, Colombia, Venezuela y México reportadas entre 2008 y 2019. Esto demuestra la dispersión de este genotipo en diferentes regiones del Perú y su relación genética evolutiva con otros países. De manera similar, el análisis filogenético del DENV-2 reveló que el genotipo cosmopolita también se dispersó en varias regiones del Perú.

Cruz C. *et al*. realizaron una investigación sobre la epidemiología molecular del DENV-2 en Perú, y sus resultados sugieren que el genotipo americano fue introducido en Perú desde antes de 2001, y que el genotipo asiático americano ha estado circulando en Perú desde 2000 en adelante ^28^, con dos linajes posiblemente introducidos desde el norte a través de países vecinos, como Ecuador, Colombia y Venezuela, y desde el este a través de Brasil y Bolivia.

Estos estudios indican que en Perú circulaban genotipos anteriores. A partir de 2022, el genotipo cosmopolita es el predominante en Perú, con una ausencia notable de otros genotipos. El análisis filogenético de los casos de DENV-2 sugiere que tras la aparición del genotipo cosmopolita en la región de Madre de Dios en 2019, este se extendió posteriormente a otras regiones del Perú, siendo posiblemente las regiones peruanas de Junín y Amazonas los centros potenciales de su transmisión temprana.

Giovanetti *et al.*^29^ investigaron la aparición del genotipo cosmopolita del DENV-2 en Brasil y descubrieron que la cepa obtenida de un paciente en Goiás está filogenéticamente relacionada con los casos de Perú y Bangladesh; lo que sugiere el inicio de una propagación regional en las Américas. Susuki K. *et al*. ^30^ encontraron dos linajes distintos de DENV-2 en Bangladesh dentro del genotipo cosmopolita. Estos linajes están estrechamente relacionados con cepas de Filipinas, Malasia y Singapur y han experimentado un aumento de la diversidad genética.

Las secuencias del genotipo cosmopolita del DENV-2 se clasifican en el linaje 5. Estas clasificaciones de linajes fueron recogidas por Yenamandra *et al.*^31^, que destacaron la gran heterogeneidad del genotipo cosmopolita y la distribución de los genotipos a lo largo del tiempo. Esto sugiere que el genotipo cosmopolita varía genéticamente con el tiempo y da paso a varios linajes que circulan en diferentes países.

Nuestros hallazgos sugieren la circulación del genotipo asiático americano en 2019 y 2020, y su posterior sustitución por el genotipo cosmopolita que surgió en 2019.

Este estudio presenta resultados importantes, pero también algunas limitaciones que pueden afectar la interpretación de estos resultados. Aunque se recolectaron muestras de varias regiones del Perú, no abarcan todas las regiones del país; lo que podría sesgar la representación de los genotipos de dengue. -2. El estudio analiza principalmente información genética de los virus Dengue, sin integrar factores clínico-epidemiológicos, lo cual restringe la evaluación del impacto de los genotipos en la gravedad de la enfermedad. Además, un análisis que integre el estudio de factores ecológicos y ambientales específicos limita podría mejorar el conocimiento de una situación más real de la enfermedad del Dengue en el Perú.

 En conclusión, los hallazgos del presente estudio demuestran la rápida dispersión y co-circulación de genotipos de DENV-1 y DENV-2 en diferentes regiones del Perú durante 2019-2022. Estas observaciones resaltan la necesidad urgente de fortalecer los sistemas de vigilancia epidemiológica y los estudios genómicos para detectar oportunamente genotipos emergentes de DENV y su potencial dispersión a otros países de las Américas.
